# Delivery of pancreatic digestive enzymes into the gastrointestinal tract by pancreatic exocrine tissue transplant

**DOI:** 10.1038/s41598-019-42362-z

**Published:** 2019-04-11

**Authors:** Kyoji Ito, Katsuhisa Matsuura, Yuichiro Mihara, Yoshihiro Sakamoto, Kiyoshi Hasegawa, Norihiro Kokudo, Tatsuya Shimizu

**Affiliations:** 1 Institute of Advanced Biomedical Engineering and Science, Tokyo Women’s Medical University, Tokyo, Japan; 2 Hepato-Biliary-Pancreatic Surgery Division, Department of Surgery, Graduate School of Medicine, The University of Tokyo, Tokyo, Japan

## Abstract

Exocrine pancreatic insufficiency, caused by disease-induced loss of pancreatic exocrine cells, may be treated through regenerative stem cell technologies that facilitate the production of pancreatic exocrine cells from induced pluripotent stem cells (iPSCs). However, delivering the digestive enzymes produced in the transplanted cells to the gastrointestinal tract remains a challenge. To generate an allogenic transplantation rat model, minced pancreas was transplanted into the gastric submucosal space with ablation of muscularis mucosa. In the allogenic transplantation, transplanted pancreatic cells were engrafted. Elevated amylase was detected in gastric juice, while transplanted cells disappeared through auto-digestion when the muscularis mucosa was not eliminated. Human iPSCs were differentiated into pancreatic exocrine cells by stage-specific treatment with growth factors and chemical compounds, and the differentiated pancreatic cells were implanted into the gastric submucosal space of nude rats. The transplanted cells were engrafted, and amylase was detected in the gastric juice in some cases. These findings suggest that transplantation of pancreatic exocrine cells into the gastric submucosal space with muscularis mucosa elimination will contribute to a regenerative approach for pancreatic exocrine insufficiency.

## Introduction

Exocrine pancreatic insufficiency is characterised by maldigestion and poor nutrition due to the insufficiency of pancreatic digestive enzymes. It is found in pancreatic diseases including cystic fibrosis and chronic pancreatitis, and following surgical resection of the pancreas and a severe attack of acute necrotising pancreatitis^1^. Clinical manifestations include abdominal cramps, steatorrhea, and malnutrition. Malnutrition caused by exocrine pancreatic insufficiency has been correlated with high morbidity and mortality secondary to an increased risk of malnutrition-related complications such as cardiovascular events^2,3^. Current treatment is based on dietary modification and oral administration of exogenous pancreatic enzymes^4,5^. However, the effectiveness is limited, and the patients must take the medicine for the rest of their lives^6^.

Recent advances in stem cell technologies have facilitated the generation of various human somatic cells from human pluripotent stem cells^7^. Several studies reported differentiation of not only pancreatic endocrine cells including pancreatic β cells^8,9^ but also pancreatic exocrine cells^10,11^ from human stem cells including embryonic stem cells and induced pluripotent stem cells (iPSCs). Many studies have already reported the recovery of pancreatic endocrine function by the transplantation of allogeneic pancreatic cells in the clinical setting and human pluripotent stem cell-derived pancreatic cells in animal models. However, there are few reports on the recovery of pancreatic exocrine function by the cell replacement approach possibly because of several issues including the transplantation site restriction and outflow tract of pancreatic enzymes. When transplanting pancreatic exocrine tissue into a patient with pancreatic exocrine insufficiency, pancreatic digestive enzymes produced in the transplanted tissue must be secreted into the upper gastrointestinal tract to achieve effective digestion^12^. The functional outflow pathway of pancreatic enzymes from the transplanted cells enables effective digestion and prevents auto-digestion of the transplanted cells and surrounding tissues^13^. However, little is known regarding effective methods of transplantation of pancreatic exocrine cells to achieve functionally appropriate delivery of pancreatic digestive enzymes from the transplanted tissue into the gastrointestinal tract.

Here, we generated an allogeneic transplantation model of rat pancreatic exocrine tissue transplanted into the gastric submucosal space to achieve functional transplantation. We also present the production of pancreatic exocrine cells from human iPSCs using a 3D bioreactor culture strategy. Using the transplantation method, we observed the engraftment of the iPSC-derived exocrine cells in the gastric submucosal space of rat.

## Results

### Transplantation of pancreas into gastric submucosal space

Pancreatic exocrine cells should be transplanted to the upper gastrointestinal tract to protect them from auto-digestion and efficient flow of pancreatic juices^12^. To achieve the functional transplantation of pancreatic exocrine tissue, we generated an allogeneic transplantation model of rat pancreatic exocrine tissue transplanted into the gastric submucosal space.

**Figure 1:**
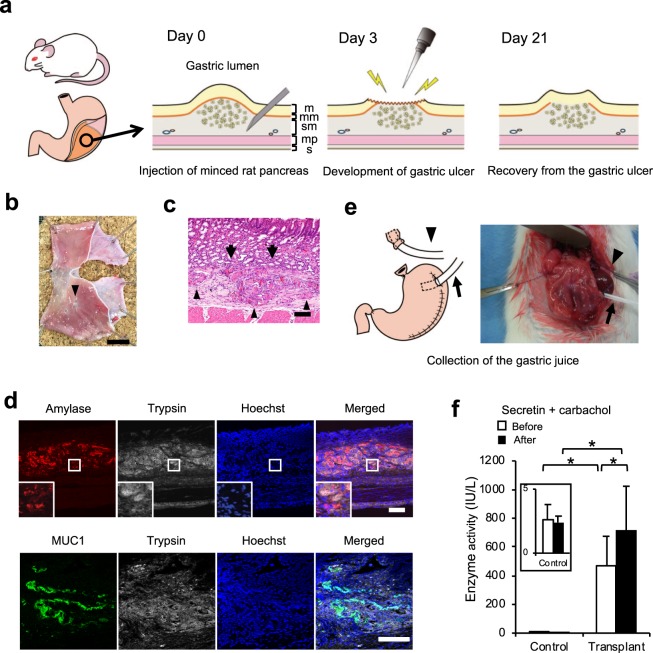
Allogenic transplantation of minced rat pancreas into the gastric submucosal space. (**a**) Scheme of the transplantation protocol. The suspension of minced pancreas was injected into the gastric submucosal space of the dorsal glandular stomach of rat under laparotomy. Three days after transplantation, the stomach was opened again under laparotomy, and mucosa and muscularis mucosa at the swelling of the transplant site were ablated with electrocautery to develop a gastric ulcer. The ulcer was treated by daily administration of proton pump inhibitor. The various structures of the gastric wall are depicted as – m: mucosa, mm: muscularis mucosa, sm: submucosa, mp: muscularis propria, s: serosa. (**b**) The stomach specimen. The gastric ulcer scar was observed on the transplant site (arrowhead). Scale bar: 10 mm. (**c**) Haematoxylin-eosin staining of the transplant site. The transplanted pancreas was observed in the gastric submucosal space (arrowhead) and directly attached to the gastric mucosa without the interference of muscularis mucosa (arrow). Scale bar: 100 μm. (**d**) Representative images of immunofluorescence staining 3 weeks after transplantation. Top: amylase (red) and trypsin (white). Bottom: MUC1 (green) and trypsin (white). Nuclei were stained with Hoechst 33258 (blue). The transplanted pancreas still expressed the acinar markers amylase and trypsin and the ductal marker MUC1. Scale bar: 100 μm. (**e**) Gastric juice was collected and the stomach was harvested 21 days after transplantation. Saliva was drained from the oral stump to outside of the abdominal cavity (arrowhead). A 16 G Surflo outer catheter was inserted into the stomach to cleanse the gastric lumen and collect the gastric juice (arrow). (**f**) Amylase level in the gastric juice. Amylase in the gastric juice was significantly elevated in the transplanted group compared to that in the control group. The administration of carbachol and secretin further increased the amylase level in the transplanted group (n = 5). All data are represented as means ± SD. *p < 0.05.

At first, the minced pancreas was injected into the gastric submucosal space of the dorsal glandular stomach under laparotomy (Fig. 1a). We observed the engraftment of the transplanted pancreas in the gastric submucosal space seven days after transplantation. However, the muscularis mucosa interfered with the efficient contact of the transplanted pancreas with the gastric lumen (Supplementary Fig. 1a). Twenty-one days after transplantation, the transplanted pancreatic cells had disappeared, and myelin figures and other membranous remnants of disintegrated cells were observed, suggesting auto-digestion of the acinar cells^14^ (Supplementary Fig. 1b) and that the elimination of muscularis mucosa might enable the engraftment of transplanted cells through the delivery of pancreatic enzymes to the gastric cavity. As previously reported^15^, both mucosa and muscularis mucosa were damaged by the gastric ulcer (Supplementary Fig. 2a), and only the mucosal layer was regenerated in the recovery process (Supplementary Fig. 2b). Therefore, we next applied this gastric ulcer healing process to the transplantation of pancreatic exocrine cells. Twenty-one days after transplantation, the gastric ulcer scar was observed on the transplant site in the specimens (Fig. 1b). The transplanted pancreas was observed in the submucosal space of the stomach (Fig. 1c), and the pancreas was directly attached to the gastric mucosa without the interference of muscularis mucosa (Fig. 1d). The transplanted pancreas still expressed acinar markers amylase and trypsin and the ductal marker MUC1 (Fig. 1d). These pancreatic exocrine markers were not detected in the control group (data not shown). Next, we collected the gastric juice before and after secretin and carbachol administration to investigate the delivery of pancreatic enzymes into the gastric cavity (Fig. 1e). We observed a low amount of amylase in the gastric juice of rats without transplantation even after secretin and carbachol stimulation. On the other hand, a high amount amylase was detected in the gastric juices in rats after transplantation and secretin and carbachol stimulation, which significantly increased the amount of amylase in the gastric juice (Fig. 1f). These findings suggested that the transplantation of pancreatic cells with muscularis mucosa elimination through the gastric ulcer healing process might enable the fabrication of functional pancreatic exocrine tissues.

### Differentiation of iPSCs into pancreatic progenitor cells

**Figure 2:**
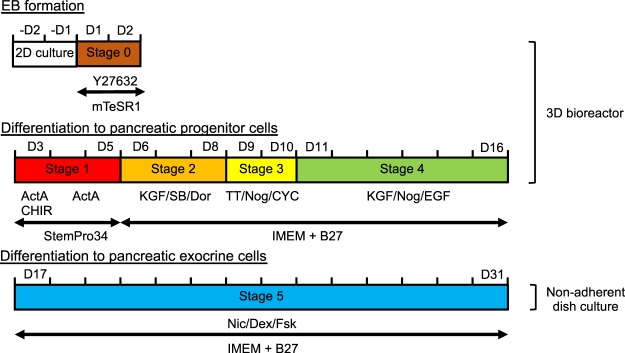
Differentiation protocol. After cell aggregation (D1–2), iPSCs were induced to differentiate following a five-stage differentiation protocol: D3–D5 (stage 1) for definitive endoderm, D6–D8 (stage 2) for primitive foregut, D9–D10 (stage 3) for posterior foregut, D11–D16 (stage 4) for pancreatic progenitors, and D17–D31 (stage 5) for pancreatic exocrine cells. ActA: Activin A, CHIR: CHIR99021, CYC: cyclopamine, D: day, Dex: dexamethasone, Dor: dorsomorphin, EB: embryonic body, EGF: epidermal growth factor, FSK: forskolin, IMEM: improved minimum essential medium, KGF: keratinocyte growth factor, Nic: nicotinamide, Nog: Noggin, SB: SB431542, TT: 4-[(E)-2-(5,6,7,8-tetrahydro-5,5,8,8-tetramethyl-2-naphthalenyl)-1-propenyl]benzoic acid.

As pancreatic exocrine cell transplantation will be necessary for patients with cystic fibrosis, chronic pancreatitis, surgical pancreatic tumour resection, and a severe attack of acute necrotising pancreatitis, autologous pancreatic tissues might not be applicable for transplantation. However, stem cell-derived pancreatic exocrine cells may be promising cell sources for transplantation. We previously reported the successful production of PDX1-positive pancreatic progenitor cells in a scalable 3D suspension bioreactor^16^, and we applied this strategy to the induction of pancreatic exocrine cells with slight modifications (Fig. 2).

**Figure 3:**
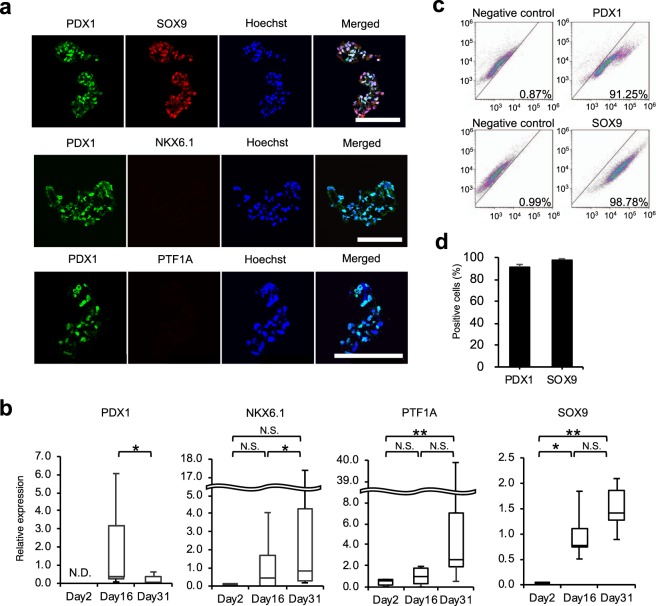
Characteristics of cell aggregates at the end of stage 4. (**a**) Representative images of immunofluorescence staining. Top: PDX1 (green) and SOX9 (red). Middle: PDX1 (green) and NKX6.1 (red). Bottom: PDX1 (green) and PTF1A (red). Nuclei were stained with Hoechst 33258 (blue). Scale bar: 100 μm. (**b**) The mRNA expression of pancreatic markers PDX1, NKX6.1, PTF1A, and SOX9 assessed by real-time polymerase chain reaction at day 2 (the end of stage 0), day 16 (the end of stage 4), and day 31 (the end of stage 5). The data are presented as the fold-change in gene expression relative to the value at day 16 and calculated from 5 (NKX6.1 and SOX9) or 7 (PDX1 and PTF1A) consecutive samples. (**c**) Representative dot plots from flow cytometry analysis. (**d**) The percentage of PDX1- and SOX9-positive cells calculated from three consecutive samples. All data are represented as means ± SD. *p < 0.05, **p < 0.01. N.D.: not detected, N.S.: not significant.

At day 16 (the end of stage 4) of our protocol, many cells in the cell aggregates expressed PDX1 as well as another pancreatic progenitor marker, SOX9, in the nuclei, while the expression of NKX6.1 and PTF1A was hardly observed. (Fig. 3a). Consistent with the immunohistochemical analysis, PDX1 and SOX9 mRNA expression was observed on day 16, while no significant up-regulation of NKX6.1 and PTF1A mRNA was observed (Fig. 3b). Flow cytometric analysis revealed that while only a few cells expressed PDX1 and SOX9 on day 2 (Supplementary Fig. 3), the fraction of PDX1^+^ and SOX9^+^ cells increased to 91.3 ± 2.4% and 98.0 ± 0.8%, respectively, on day 16 (Fig. 3c,d) . These findings suggest that human iPSCs differentiated into early pancreatic progenitor cells.

### Differentiation of iPSCs into pancreatic exocrine cells

Following stage 4, cells were transferred into non-adherent plates and cultured for a further 2 weeks in the presence of nicotinamide, dexamethasone, and forskolin (Fig. 2).

**Figure 4:**
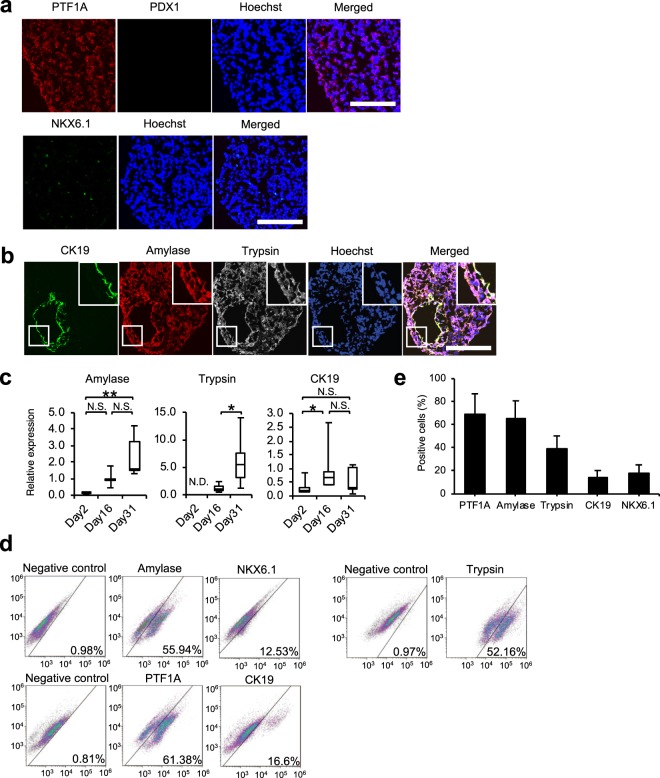
Characteristics of cell aggregates at the end of stage 5. (**a**) Representative images of immunofluorescence staining of cell aggregates. Top: PTF1A (red) and PDX1 (green). Bottom: NKX6.1 (green). Nuclei were stained with Hoechst 33258 (blue). Scale bar: 100 μm. (**b**) Representative images of immunofluorescence staining for CK19 (green), amylase (red), and trypsin (white) expression of cell aggregates. Nuclei were stained with Hoechst 33258 (blue). Scale bar: 100 μm. (**c**) The mRNA expression of pancreatic acinar markers amylase and trypsin and pancreatic ductal marker CK19 assessed by real-time polymerase chain reaction at day 2 (the end of stage 0), day 16 (the end of stage 4), and day 31 (the end of stage 5). The data are presented as the fold-change in gene expression relative to the value at day 16 and calculated from five consecutive samples. (**d**) Representative dot plots from flow cytometry analysis. The same negative control was used for amylase and NKX6.1, and PTF1A and CK19. (**e**) The percentage of PTF1A-, amylase-, trypsin-, CK19-, and NKX6.1-positive cells calculated from three consecutive samples. All data are represented as means ± SD. *p < 0.05, **p < 0.01. N.D.: not detected; N.S.: not significant.

mRNA expression of PDX1 was significantly down-regulated at day 31, while NKX6.1, PTF1A, and SOX 9 were remarkably expressed at day 31 (Fig. 3b), suggesting that the pancreatic progenitor cells at day 16 further differentiated into pancreatic cells. In line with the results of PCR analysis, PTF1A expression, but not that of PDX1, was clearly observed in many cells in cell aggregates at day 31 (Fig. 4a). Some endocrine lineage cells expressing NKX6.1 and glucagon and a small number of albumin-expressing cells were observed (Fig. 4a, Supplementary Fig. 4a,b). On the other hand, pancreatic acinar cells expressing amylase and trypsin were observed in many cells in cell aggregates, while some ductal cells expressing CK19 were also observed (Fig. 4b). Amylase and trypsin mRNA expression levels at day 31 were significantly up-regulated compared with those at day 2 or day 16, while mRNA expression levels of CK19 were remarkably up-regulated at day 16 compared to those at day 2, and the expression levels were maintained until day 31 (Fig. 4c). However, mRNA expression levels of acinar markers in iPSC-derived pancreatic exocrine cells were lower compared to those in adult pancreas (Supplementary Fig. 5), indicating an immature status of the differentiated pancreatic acinar cells. Consistent with the significant up-regulation of mRNA expression levels of acinar and ductal markers at day 31 compared to that at day 2, many cells expressed PTF1A (69.4 ± 17.7%), amylase (64.9 ± 15.9%), trypsin (39.6 ± 10.8%), CK19 (14.8 ± 5.6%), and NKX6.1 (14.8 ± 5.6%) at day 31, but not at day 2 (Fig. 4d,e, Supplementary Fig. 3). Collectively, these results suggest that the human iPSCs might differentiate chiefly into acinar and ductal lineage cells.

### Implantation of iPSC-derived pancreatic exocrine cells

**Figure 5:**
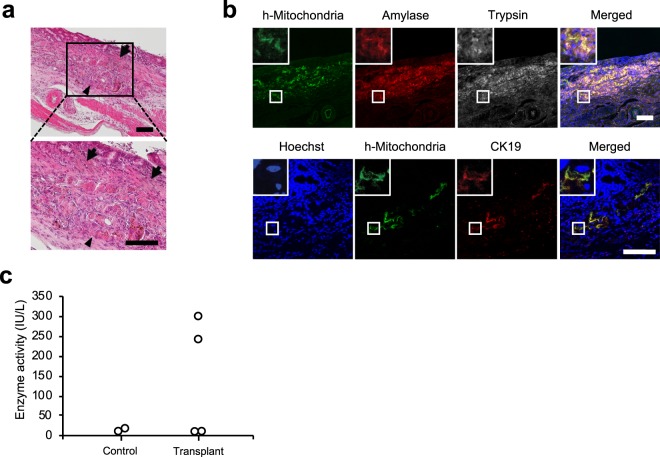
Implantation of iPSC-derived pancreatic exocrine cells into the gastric submucosal space of rat. (**a**) Haematoxylin-eosin staining of the implanted cells. The implanted cells were observed in the submucosal space of the stomach (arrowhead), and the implanted pancreas directly attached to the gastric mucosa without the interference of muscularis mucosa (arrow). Scale bar: 100 μm. (**b**) Representative images of immunofluorescence staining at the implantation site. Top: human mitochondria (green), amylase (red), and trypsin (white). Bottom: human mitochondria (green) and cytokeratin 19 (red). Nuclei were stained with Hoechst 33258 (blue). The implanted iPSC-derived exocrine pancreas still expressed amylase and trypsin. Scale bar: 100 μm. (**c**) Amylase level in the gastric juice. Amylase in the gastric juice was elevated in two out of four implanted rats, while there was no elevation in the other 2 rats (n = 4). The elevation of amylase was not detected in the gastric juice of the control group (n = 2).

Using the same procedure as that of the allogeneic transplantation of minced rat pancreas (Fig. 1a), we implanted iPSC-derived pancreatic exocrine cells into the gastric submucosal space of nude rats (n = 4). Twenty-one days after implantation, we observed acinar-like tissues in the submucosal space of the stomach, and the tissue directly attached to the gastric mucosa without the interference of muscularis mucosa (Fig. 5a). Immunofluorescence analysis revealed the expression of CK19, amylase, and trypsin along with human mitochondria in the tissue (Fig. 5b), suggesting that the tissue derived from implanted iPSC-derived pancreatic exocrine cells might be engrafted without auto-digestion. We observed the engraftment of the implanted iPSC-derived pancreatic exocrine cells in three out of four implanted rats (Supplementary Fig. 6). Elevated amylase in the gastric juice was observed in 2 of 4 implanted rats, while the amylase levels in other 2 rats were comparable to those in the control (Fig. 5c).

## Discussion

Pancreatic exocrine insufficiency is caused by the shortage of pancreatic exocrine enzymes for food digestion, and the symptoms are developed when postprandial secretion of pancreatic enzymes is decreased below 10% of the normal level. Malnutrition caused by pancreatic exocrine insufficiency increases the risk of malnutrition-related complications and cardiovascular events^2^. In fact, pancreatic exocrine insufficiency associated with chronic pancreatitis increases the risk of death 4 to 5 times compared to that of the general population matched by age and gender^3^. Current therapy for pancreatic insufficiency is based on the oral administration of exogenous pancreatic enzymes and dietary modifications. A recently published randomised, double-blind, placebo-controlled trial in chronic pancreatitis patients has shown the significant therapeutic efficacy of modern enzyme preparations in reducing fat excretion, decreasing stool frequency, and improving stool consistency^4^. Similar efficacy was shown in a placebo-controlled trial in patients suffering from cystic fibrosis with pancreatic exocrine insufficiency and steatorrhea^5^. However, despite the use of modern enteric-coated enzyme preparations in minimicrospheres, fat digestion does not revert to normal in almost half of the patients with pancreatic exocrine insufficiency^6^. Moreover, the patients must take the enzyme substitution therapy for the rest of their lives. Although the production of pancreatic exocrine cells from iPSCs was recently reported^10,11^, a regenerative approach to pancreatic exocrine insufficiency using stem cell-derived pancreatic exocrine cells has not yet been studied. Direct replacement of pancreatic enzymes by a regenerative approach could solve the limitations of the current drug substitution therapy and enhance the quality of life of the patients.

Achieving safe and functional transplantation of exocrine cells is a difficult challenge, as incomplete exocrine drainage into the intestine would lead to harmful accumulation of exocrine enzymes in the surrounding tissues^12^. To achieve functional transplantation of pancreatic exocrine tissue, we injected minced rat pancreas into the gastric submucosal space of rats and later developed a gastric ulcer to eliminate muscularis mucosa, which inhibits the delivery of enzymes from the transplanted pancreas to the gastric cavity. After transplantation, we detected the basal secretion of amylase in the gastric juice before secretin and carbachol stimulation^17^. The administration of secretin and carbachol further elevated amylase, suggesting the efficient functioning of the transplanted pancreatic exocrine, although pancreatic enzymatic activity found in the gastric juice after stimulation was still low compared with several hundred thousand units/litter of amylase activity in human pancreatic juice. Further studies on the responses against dose-dependent effects of secretin or carbachol and on the multiple transplantation to several sites will contribute to achieve clinically effective enzymatic activity in the gastric juice after pancreatic exocrine cell transplantation. However, to the best of our knowledge, this is the first study to achieve functional transplantation of pancreatic exocrine tissues into the gastrointestinal tract without suturing the pancreatic duct. Although the acidic environment of the gastric lumen might affect the activity of pancreatic enzymes^18^, the gastric wall could be a reasonable site for the transplantation of pancreatic exocrine tissue from a clinical application perspective. The stomach is easy to approach by endoscope for transplantation and subsequent observation, and unexpected events such as tumour formation or obstructive pancreatitis may be easily addressed. Although gastric inactivation of pancreatic enzymes derived from the transplanted cells through low pH is one of concerns, the administration of a proton pump inhibitor will prevent the inactivation since it has been reported to increase gastric pH over 6^19^. Further studies regarding the appropriate number of cells for transplantation based on the quantitative evaluation of pancreatic enzyme activities in the acidic gastric environment are required. In addition, as the structure of the intestinal wall is the same as that of the stomach, our transplantation procedure could be applied to the intestinal tracts including duodenum. The transplantation to duodenum may avoid the pancreatic enzyme inactivation through low pH, while multiple site transplantation might be necessary because the amount of cells which can be transplanted per site will be much smaller than that of the stomach.

Recent advances in differentiating stem cells to pancreatic cells have enabled the production of both endocrine^8,9^ and exocrine^10,11^ pancreatic cells from embryonic stem cells or iPSCs. PDX1 is an essential transcription factor for the development of the pancreas, and PDX1-expressing cells are known to be pancreatic progenitor cells that have a potential to differentiate into both endocrine and exocrine cells^20^. During differentiation, the expression of PDX1 is down-regulated in the exocrine cells, while the high expression levels are maintained in β cells of the endocrine lineage^21^. NKX6.1 and PTF1A are other essential transcription factors for pancreas development. NKX6.1 expression is required for the differentiation of mature endocrine pancreas^22^, and PTF1A expression contributes to the formation of acinar cells of the exocrine lineage^23,24^. NKX6.1 and PTF1A become mutually exclusive while the endocrine and exocrine lineages develop^25^. We previously reported the substantial production of pancreatic progenitor cells using a 3D bioreactor. At the end of differentiation, we observed a diffuse expression of trypsin in the cytoplasm of the produced cells^16^. In the present study, more than 90% of the differentiated cells were PDX1 positive at day 16. The mRNA expression of PDX1 was highest at day 16 and was subsequently down-regulated at day 31. At day 31, the pancreatic acinar cell markers including amylase and trypsin and the ductal marker CK19 were also expressed. In addition, we observed a small number of endocrine lineage cells expressing NKX6.1 and glucagon. Although several reports have already shown the differentiation of pancreatic acinar and ductal cells (Hohwieler *et al*.^11^ reported the differentiation of about 30% acinar cells and 60% ductal cells; Huang *et al*.^10^ reported the differentiation of about 1% acinar cells and 10% ductal cells), the present study showed more efficient differentiation of pancreatic acinar and ductal cells (around 40–60% and 15%, respectively). Compared to our previous protocol, the absence of Alk5 inhibitor II, which inhibits TGF-β type I receptor and induces pancreatic endocrine differentiation effectively^26,27^, in stage 5 cells might contribute to the differentiation to exocrine cells rather than endocrine cells. The differences in the iPSC lines used might have contributed to the differences in the efficiency of their differentiation to pancreatic endocrine and exocrine cells. It has been reported that pancreatic endocrine differentiation efficiency is different among several human pluripotent stem cell lines^28^. We previously reported the successful differentiation of PDX1/NKX6.1 positive pancreatic endocrine progenitor cells^16^ (253G1 cell line); however, very few NKX6.1 positive cells were observed in the present study (201B7 cell line). Despite the evidence of protein expression of amylase and trypsin in cell aggregates at day 31, the pancreatic exocrine enzymes from these cells were not observed in the culture supernatant even after stimulation with secretin and carbachol *in vitro* (data not shown), suggesting that the maturation levels were not sufficient for pancreatic enzyme secretion. The mRNA expression levels of acinar markers in iPSC-derived pancreatic exocrine cells, which were lower than those in adult pancreas, support the premature status of the differentiated cells as pancreatic exocrine cells. On the other hand, we detected the elevation of amylase in the gastric juice in two out of four rats 21 days after implantation of iPSC-derived pancreatic exocrine cells, suggesting that an *in vivo* environment might promote maturation of the differentiated pancreatic exocrine cells. The mesenchyme around developing pancreatic cells has been reported to play an important role in acinar differentiation. The cell aggregates after differentiation might not contain a sufficient number of mesenchymal cells, as over 90% of cells were PDX1- and SOX9-positive pancreatic progenitor cells at the end of stage 4, while a few mesenchymal cells exist in the gastric submucosal space. Therefore, mesenchymal cells around the transplanted pancreatic exocrine cells might affect their maturation. In addition, a relative deficiency of pancreatic enzymes is found in new-borns compared with that in adults, and maturation of the pancreatic exocrine function is promoted after birth^29^, suggesting that exocrine pancreas maturation might involve digestion. The maturation of implanted iPSC-derived pancreatic exocrine cells might be accelerated by implantation into the stomach and participation in digestive function. To support this hypothesis, further studies on the differences in gene expression profiles in these cells before and after implantation using RNA sequencing analyses are required.

Using the transplantation procedure, we observed the engraftment of iPSC-derived exocrine cells in the submucosal space of the rat stomach. However, transplanted iPSC-derived pancreatic exocrine cells were not observed in a rat and amylase was not detected in the gastric juice in two rats. Since allogeneic transplanted rat pancreatic exocrine cells were observed and amylase was also detected in the gastric juice in all cases, the transplantation procedure itself might be effective and immunorejection due to xenotransplantation might be one of the reasons for the insufficient engraftment and the variability of enzyme detection between samples. Nude rats in the present study were not appropriate for sufficient engraftment. Using immunodeficient animals such as the NOG mice and treatment with immunosuppressive drugs will promote engraftment of the transplanted iPSC-derived exocrine cells and provide information on the time-dependent maturation and functional profile of the implanted cells.

Our transplantation procedure might be applicable to other organs with an exocrine function such as liver, as bile juice can be drained into the gastrointestinal tract. Although current progress in cell differentiation techniques has facilitated the generation of liver cells from stem cells^30^, the functional transplantation, namely methods to produce the outflow of excretion from the generated cells, remains an unsolved problem. Our transplantation procedure could contribute to a regenerative approach for organs with exocrine function.

There were some limitations to this study. We only used one iPSC line for the differentiation of iPS cells to pancreatic exocrine cells in this study. Therefore, it remains unclear whether the differentiation protocol used in the present study may be applied to pancreatic exocrine differentiation in other iPSC lines. Further refinement of culture conditions will be necessary for clinical-grade iPSCs for regenerative medicine. We did not apply any purification strategies before transplantation. Although no purification was performed, we did not find any tumours at 21 days after transplantation. The main purpose of this study was to fabricate functional pancreatic exocrine tissues by transplantation into the gastrointestinal tract. Further development will be necessary to prevent tumour formation upon transplantation by eliminating the remaining iPSCs.

In conclusion, we achieved functional allogenic transplantation of pancreatic exocrine tissue into the gastric submucosal space of rats. Using the transplantation procedure, iPSC-derived pancreatic exocrine cells were engrafted in the gastric submucosal space, and the digestive enzymes produced in the transplanted tissue were delivered into the gastrointestinal tract.

## Methods

### Cultivation of undifferentiated iPSCs

The human iPSC 201B7 strain^7^ was purchased from RIKEN (Tsukuba, Japan) and maintained in Primate ES Cell Medium (ReproCELL, Yokohama, Japan) supplemented with 5 ng/mL basic fibroblast growth factor (ReproCELL) on mitomycin C-treated mouse embryonic fibroblasts (ReproCELL) at 37 °C in a humidified atmosphere with 5% CO_2_. Cells were passaged as small clumps every 3–4 days using dissociation solution for human ESCs/iPSCs (ReproCELL).

### Transplantation of pancreas into gastric submucosal space

Animal studies were performed according to ARRIVE guidelines. All animals were housed two per cage in a temperature-, humidity-, and light-controlled room. Food and water were available *ad libitum*. After transplantation, animals were individually housed. In all experiments, animals were randomly assigned to the experimental groups. All animal experiments were approved by the Ethics Committee for Animal Experimentation of Tokyo Women’s Medical University and performed according to the ‘Guide for the Care and Use of Laboratory Animals’ published by the US National Institutes of Health (NIH publication No. 85–23, revised 2011).

The left half of the pancreas was removed from male F344/Jcl rats aged 8–10 weeks, rinsed with Hank’s balanced salt solution with calcium and magnesium (Nakalai Tesque, Kyoto, Japan) three times, and minced. The minced pancreatic fragments were suspended into Hank’s balanced salt solution and filtered through one layer of gauze to collect the small fragments. The size of the fragments of the minced rat pancreas was less than 500 μm. The collected fragments were rinsed three times, resuspended in Hank’s balanced salt solution to approximately 5 × 10^6^ cells/200 μL, and kept on ice.

The pancreatic fragments were transplanted into the gastric submucosal space of male F344/Jcl rats aged 8–10 weeks. After a 24-h fast, rats were anaesthetised with isoflurane inhalation. Under laparotomy, the stomach was mobilized from surrounding organs, and a 1 cm incision was made on the greater curvature of the forestomach. After washing the internal lumen of the stomach with normal saline, a 200-μL suspension of the minced pancreas (5 × 10^6^ cells) was injected into the gastric submucosal space of the dorsal glandular stomach. After suturing the opened stomach, the stomach was returned to the original place, and the wound was closed.

Three days after transplantation, the stomach was opened again under laparotomy, and a gastric ulcer was developed on the swelling of the transplant site using electrocautery by ablating the mucosa and muscularis mucosa. After suturing the opened stomach, the stomach was returned to the original place, and the wound was closed. Lansoprazole at 10 mg·kg^−1^·day^−1^ was administered to the rats everyday beginning from the day after the development of the gastric ulcer till the day of sacrifice.

At 21 days post-transplantation, gastric juice was collected. After laparotomy and mobilization of the stomach, the oesophagus was ligated and cut just above the cardia of the stomach, and the duodenum was ligated just distal to the pylorus to prevent contamination of the gastric juice with saliva and duodenum fluid. The saliva was drained from the oral stump of the oesophagus to the outside of the abdominal cavity with a 20 G Surflo outer catheter (Terumo, Tokyo, Japan) to avoid contamination of the abdominal cavity. To cleanse the gastric lumen, a 16 G Surflo outer catheter was inserted into the forestomach, and the lumen was irrigated with normal saline until the cleanse fluid became completely clear. After removing the normal saline, the abdominal cavity was temporarily closed for 2 h, and the gastric juice was collected from the catheter inserted into the gastric lumen. After the first collection of gastric juice, the abdominal cavity was opened again, and carbachol (1 µmol/kg) and secretin (100 nmol/kg) were injected into the inferior vena cava. Again, the abdominal cavity was closed temporarily, and gastric juice was sampled 2 h later. After sampling gastric juice, the duodenum was cut, and the stomach was harvested. Amylase was quantified using routine laboratory methods (Nagahama Life Science Laboratory, Shiga, Japan). Briefly, amylase was measured by detecting p-nitrophenol which is a derivative of p-nitrophenyl 6(5)-O-benzyl-alpha-maltopentaoside after digestion by amylase. Therefore, the digestive activity of alfa-amylase was measured. This method detects both rat amylase and human amylase. The control group of rats underwent the same procedure as mentioned above and 200-μL Hank’s balanced salt solution without any cells was injected into the submucosal space of the gastric wall. Lansoprazole was also administered after surgery as mentioned above.

### 3D culture of human iPSCs for pancreatic differentiation

For differentiation, human iPSC aggregates (approximately 1 × 10^7^ cells) from three 2D culture dishes (10-cm diameter) were collected using dissociation solution and resuspended in 30 mL mTeSR1 medium (Stem Cell Technologies, Vancouver, Canada) containing 10 µM Y27632 (Wako, Tokyo, Japan) and seeded in a stirred 30-mL 3D bioreactor. The agitation rate was 55 rpm.

After cultivation for 2 days, cell aggregates were cultured in StemPro34 medium (Thermo Fisher Scientific, Waltham, MA, USA) containing 50 µg/mL ascorbic acid (Sigma–Aldrich, St. Louis, MO, USA), 2 mM L-glutamine, and 400 µM 1-thioglycerol (Sigma–Aldrich) for stage 1 (days 3–6) and subsequently in improved minimum essential medium (Thermo Fisher Scientific) containing 1% B27 Supplement (Thermo Fisher Scientific), 1% penicillin/streptomycin, and 400 µM monothioglycerol (Sigma–Aldrich) for stages 2–5 (days 6–31). The cells were differentiated with the bioreactor until stage 4 and transferred into a HydroCell plate (CellSeed, Tokyo, Japan) at stage 5. The following growth factors and small molecules were added at the corresponding days – day 3 (stage 1): 100 ng/mL Activin A (R&D Systems, Minneapolis, MN, USA) and 3 μM CHIR 99021 (TOCRIS Bioscience, Bristol, UK); days 4–5: 100 ng/mL Activin A; days 6–8 (stage 2): 50 ng/mL keratinocyte growth factor (KGF) (Peprotech, Rocky Hill, NJ, USA), 10 mM SB431542 (TOCRIS), and 1 μM dorsomorphin (Calbiochem, La Jolla, CA, USA); days 9–10 (stage 3): 3 nM 4-[(E)-2-(5,6,7,8-tetrahydro-5,5,8,8-tetramethyl-2-naphthalenyl)-1-propenyl]benzoic acid (Sigma–Aldrich), 2.5 μM cyclopamine (Sigma–Aldrich), and 50 ng/mL Noggin (StemRD, Burlingame, CA, USA); days 11–16 (stage 4): 50 ng/mL KGF, 50 ng/mL Noggin, and 50 ng/mL epidermal growth factor (Peprotech); days 17–31 (stage 5)^16^: 10 μM forskolin (FOCUS Biomolecules, Plymouth Meeting, PA, USA), 10 mM nicotinamide (PhytoTechnology Laboratories, Overland Park, KS, USA), and 10 μM dexamethasone (Wako). The cells were maintained at 37 °C in a humidified atmosphere with 5% CO_2_. For stages 2 and 3, the medium was exchanged only on the first day of each stage, and for stages 4 and 5, the medium was exchanged every 2 and 3 days, respectively.

### Implantation of iPSC-derived pancreatic exocrine cells

The cell aggregates (100–500 μm) of iPSC-derived pancreatic exocrine cells were resuspended in stage 5 medium to approximately 5 × 10^6^ cells/200 μL and kept on ice; 200 μL of the suspension was implanted in male F344/rnu rats aged 8–10 weeks. The implantation of the cells, ablation of the transplant site, collection of the gastric juice, and harvest of the stomach were performed using the same procedure as that was used for the allogeneic transplantation of minced rat pancreas into the gastric submucosal space mentioned above (Transplantation of pancreas into gastric submucosal space section). The control group rats underwent the same procedure as mentioned above and 200 μL stage 5 medium without any cells was injected into the submucosal space of the gastric wall.

### Real-time reverse transcription-polymerase chain reaction (PCR)

Total RNA was isolated from differentiated cells using an RNeasy Micro Kit (QIAGEN, Venlo, the Netherlands), and human pancreas total RNA (Takara Bio USA, Mountain View, CA, USA) was used for the comparison of the expression of pancreatic genes. cDNA was synthesised using a High Capacity cDNA Reverse Transcription Kit (Applied Biosystems, Stockholm, Sweden) with random hexamer primers. Real-time PCR analysis of each sample was then performed with a StepOne and StepOnePlus Real-Time PCR System (Applied Biosystems). TaqMan assays for real-time PCR (Applied Biosystems) are listed in Supplementary Table 1.

### Immunofluorescence staining

Cells were fixed with 4% paraformaldehyde for 30 min before embedding in OCT compound (Sakura, Tokyo, Japan) and subsequent snap-freezing. Tissue samples were fixed with 4% paraformaldehyde for 48 h, processed for paraffin embedding, and deparaffinised before preblocking. Immunofluorescence staining was carried out on serial sections of 5 μm thickness. Samples were preblocked with phosphate-buffered saline (PBS) containing 2% donkey serum, 2% bovine serum albumin (BSA), and 0.2% NP-40 for 30 min at room temperature. Primary and secondary antibodies were diluted with PBS containing 2% donkey serum, 2% BSA, and 0.1% NP-40. Primary antibodies were applied overnight at 4 °C and are summarised in Supplementary Table 2. Following three washes with PBS, secondary antibodies were applied to visualise primary antibodies for 60 min at room temperature (protected from light). Fluorescence labelling was performed using Alexa Fluor 488, 594, and 647 secondary antibodies (Thermo Fisher Scientific, all diluted 1:300). Following three washes with PBS, the slides were mounted with Fluoromount (Sigma–Aldrich). Images were acquired using a laser confocal microscope (Olympus, Tokyo, Japan).

### Flow cytometric analysis

Cellular aggregates were dissociated with Accumax (Innovative Cell Technologies Inc., San Diego, CA, USA) for 10 min at 37 °C, followed by pipetting to separate the cells. Following three washes with PBS, cells were fixed with 4% paraformaldehyde for 30 min and preblocked with PBS containing 5% BSA and 0.2% NP-40. Primary antibodies (Supplementary Table 2) were diluted with preblocking buffer and applied for 60 min. Following three washes with PBS, secondary antibodies diluted in preblocking buffer were applied for 60 min at room temperature (protected from light). Fluorescence labelling was performed using Alexa Fluor 488 and 647 secondary antibodies (Thermo Fisher Scientific, all diluted 1:100). Following three washes with PBS, stained cells were analysed using Gallios and Kaluza software (Beckman Coulter, Brea, CA, USA). Cells incubated with isotype control antibodies corresponding to each primary antibody and subsequently incubated with fluorochrome tagged secondary antibodies were used as the negative control.

### Statistical analyses

Data are presented as the mean ± standard deviation or median with interquartile ranges. Amylase value in gastric juice was assessed using unpaired and paired Student’s *t*-tests. Analysis of gene expression during differentiation was performed using the Wilcoxon signed-rank test or Friedman’s test with Scheffé's method, or one factor repeated measures ANOVA with Bonferroni’s method according to the distribution of the data. Values of *p* < 0.05 were considered significant.

## Supplementary information

Supplementary Information

## Data Availability

The data that support the findings of this study are available from the corresponding author, (K.M), upon reasonable request.
