# Supplementary material for: Delivery of pancreatic digestive enzymes into the gastrointestinal tract by pancreatic exocrine tissue transplant
Source: Sci Rep. 2019 Apr 11;9:5922. doi: 10.1038/s41598-019-42362-z (PMC6459827; doi:10.1038/s41598-019-42362-z)
Supplement: Supplementary file 1 — Supplementary Information [file 41598_2019_42362_MOESM1_ESM.pdf]

Title: Delivery of pancreatic digestive enzymes into the gastrointestinal tract by pancreatic exocrine tissue transplant

Short title: Pancreatic exocrine tissue transplant

Kyoji Ito[1, 2], Katsuhisa Matsuura[1*], Yuichiro Mihara[1,2], Yoshihiro Sakamoto[2], Kiyoshi Hasegawa[2], Norihiro Kokudo[2] and Tatsuya Shimizu[1]

[1]Institute of Advanced Biomedical Engineering and Science, Tokyo Women's Medical University, Tokyo, Japan

[2]Hepato-Biliary-Pancreatic Surgery Division, Department of Surgery, Graduate School of Medicine, The University of Tokyo, Tokyo, Japan

***Correspondence to:** Katsuhisa Matsuura, Institute of Advanced Biomedical Engineering and Science, Tokyo Women's Medical University, 8-1 Kawada-cho, Shinjuku-ku, Tokyo, 162-8666, Japan.

Tel: +81-3-5367-9945; Fax: +81-3-3359-6046

E-mail: matsuura.katsuhisa@twmu.ac.jp

SREP-18-25817-T-R1

Supplementary Table 1. List of primers used for Applied Biosystems PCR assay.

Gene	Applied Biosystems TaqMan assay ID
PDX1	Hs00236830_m1
NKX6-1	Hs00232355_m1
PTF1A	Hs00603586_g1
SOX9	Hs00165814_m1
AMY2A	Hs00420710_g1
PRSS58	Hs00976361_m1
KRT19	Hs00761767_s1
GAPDH	Hs00266705_g1

Supplementary Table 2. Antibodies applied for flow cytometry and immunostaining (of cells and organoid cryo/paraffin sections).

Antigen	Species	Cat. No.	Company	Dilution
PDX1	Goat	AF2419	R&D Systems	200: 1
NKX6.1	Rabbit	HPA036774	Atlas Antibodies	100: 1
PTF1A	Mouse	ab57257	Abcam	300: 1
SOX9	Rabbit	AB5535	EMD Millipore	100: 1
CK19	Mouse	M0888	Dako	200: 1
MUC1	Rabbit	ab45167	Abcam	500: 1
Pancreatic alpha amylase	Rabbit	ab21156	Abcam	400: 1
Trypsin	Sheep	AF3848	R&D Systems	200: 1
Glucagon	Mouse	MAB8497	R&D Systems	100: 1
C-peptide	Rabbit	GTX124265	GENETEX	100: 1
Albumin	Goat	A80-129A	Bethyl Laboratories	200: 1
Human mitochondria	Mouse	MAB1273	EMD Millipore	50: 1

Supplementary Figure 1

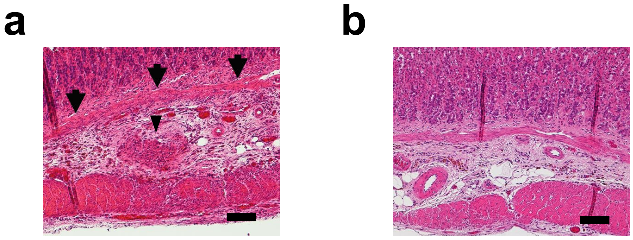

Supplementary Figure 2

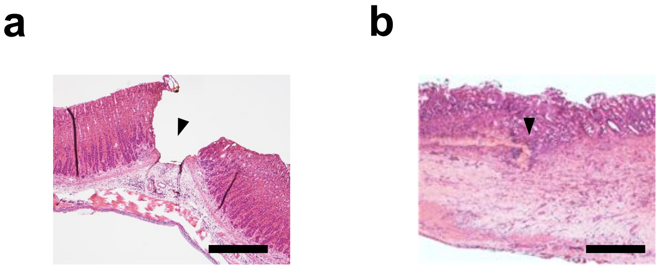

Supplementary Figure 3

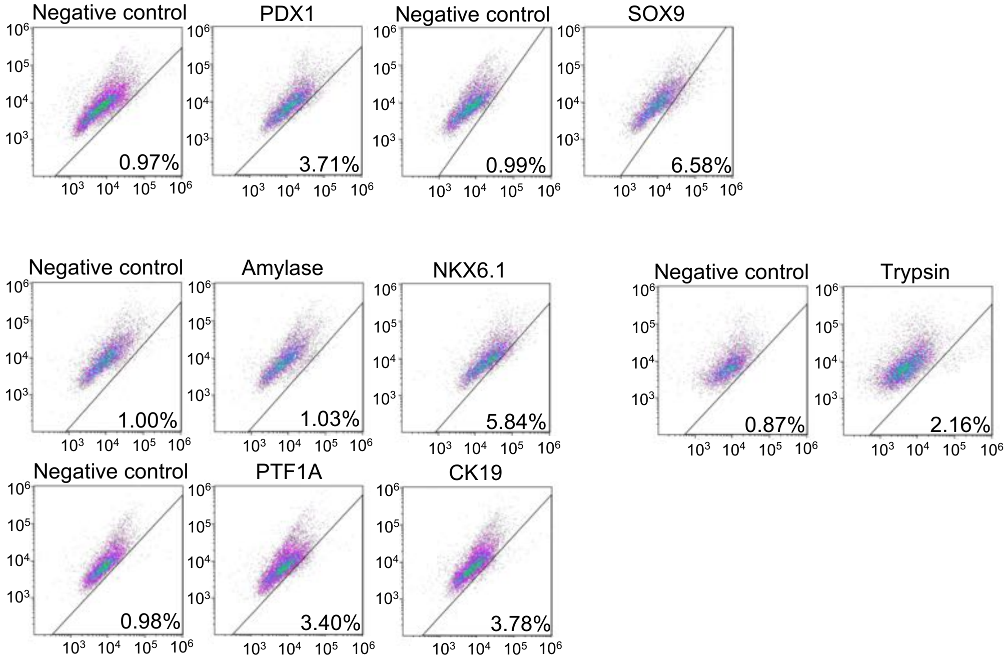

Supplementary Figure 4

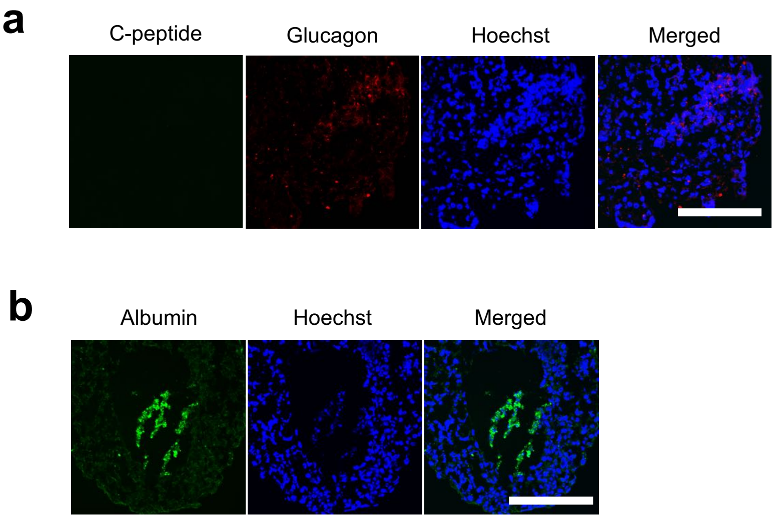

Supplementary Figure 5

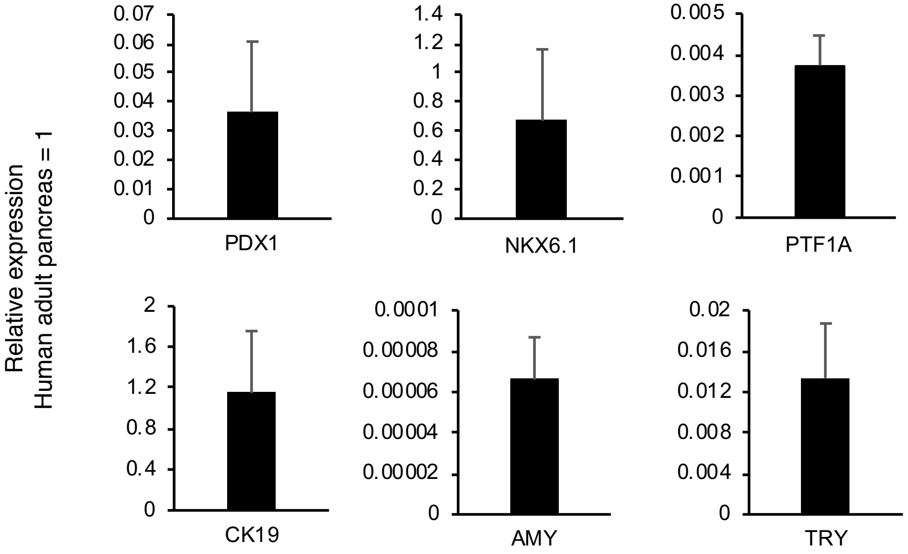

Supplementary Figure 6

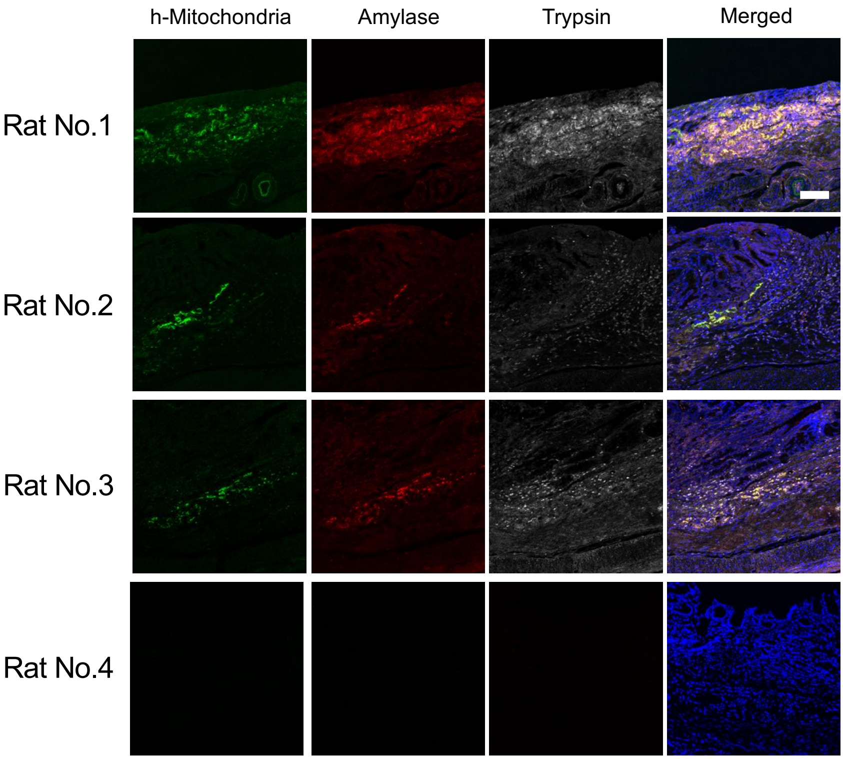

Supplementary Figure 1. Allogenic transplantation of minced rat pancreas into the gastric submucosal space. (**a**) Haematoxylin-eosin staining of the transplanted pancreas 7 days after transplantation. The transplanted pancreas was observed in the submucosal space of the stomach (arrowhead). Muscularis mucosa interfered with the contact of the transplanted pancreas to the gastric lumen (arrow). Fat necrosis was observed around the transplanted cells. (**b**) Haematoxylin-eosin staining of the transplantation site 21 days after transplantation. The transplanted pancreas vanished, and fat necrosis with saponification and surrounding fibrosis resulting from inflammatory reaction were observed, suggesting the auto-digestion of the transplanted pancreas. Scale bar: 500 μm.

Supplementary Figure 2. Evaluation of muscularis mucosa in gastric ulcer. (**a**) Haematoxylin-eosin staining of the gastric wall 3 days after the development of gastric ulcer by electrocautery. Both mucosa and muscularis mucosa were damaged by the gastric ulcer (arrowhead). (**b**) Haematoxylin-eosin staining of the gastric wall after healing of the gastric ulcer. The regeneration occurred only in the mucosal layer, and muscularis mucosa was disrupted after recovery of the gastric ulcer (arrowhead). Scale

bar: 500 µm.

Supplementary Figure 3. Representative dot plots from flow cytometry analysis at day 2.

Supplementary Figure 4. Immunofluorescence staining of cell aggregates at the end of stage 5 for (**a**) C-peptide (green) and glucagon (red) and (**b**) albumin (green). Nuclei were stained with Hoechst 33258 (blue). Scale bar: 100 µm.

Supplementary Figure 5. Comparison of mRNA expression of pancreatic markers between the adult pancreas and iPSC-derived pancreatic exocrine cells (n = 3). The levels of acinar markers in iPSC-derived pancreatic exocrine cells were lower than those in the adult pancreas.

Supplementary Figure 6. Immunofluorescence staining of the implantation site of all four rats. Human mitochondria (green), amylase (red), and trypsin (white). Nuclei were

stained with Hoechst 33258 (blue). The implanted cells were observed in Rat No. 1 and 3, a few implanted cells were observed in Rat No. 2 and no implanted cells were observed in Rat No. 4. Scale bar, 100 μm.
